# Sensory cognitive abnormalities of pain in autism spectrum disorder: a case–control study

**DOI:** 10.1186/s12991-016-0095-1

**Published:** 2016-03-05

**Authors:** Yuka Yasuda, Ryota Hashimoto, Aya Nakae, Hongling Kang, Kazutaka Ohi, Hidenaga Yamamori, Michiko Fujimoto, Satoshi Hagihira, Masatoshi Takeda

**Affiliations:** Department of Psychiatry, Osaka University Graduate School of Medicine, D3, 2-2, Yamadaoka, Suita, Osaka, 565-0871 Japan; Molecular Research Center for Children’s Mental Development, United Graduate School of Child Development, Osaka University, D3, 2-2, Yamadaoka, Suita, Osaka, 565-0871 Japan; Department of Anesthesiology and Intensive Care Medicine, 2-2, Yamadaoka, Suita, Osaka, 565-0871 Japan; Immunology Frontier Research Center, BioSystems Building, Osaka University, 1-3, Yamadaoka, Suita, Osaka, 565-0871 Japan

**Keywords:** Autism spectrum disorder (ASD), Sensory abnormality, Pain processing, Quality of pain, Visual Analog Scale (VAS), Short-form McGill Pain Questionnaire (SF-MPQ)

## Abstract

**Background:**

The Diagnostic and Statistical Manual of Mental Disorders, 5th Edition (DSM-5) recently included sensory processing abnormalities in the diagnostic criteria for individuals with autism spectrum disorder (ASD). However, there is no standard method for evaluating sensory abnormalities in individuals with ASD.

**Methods:**

Fifteen individuals with ASD and 15 age- and sex-matched controls were enrolled in this study. We compared objective pain sensitivity by measuring the pain detection threshold and pain tolerance to three different stimuli (electricity, heat, and cold). Then, we compared both subjective pain sensitivity, assessed by the visual analog scale (VAS), and quality of pain, assessed by the short-form McGill Pain Questionnaire (SF-MPQ), to determine the maximum tolerable pain intensities of each stimulation.

**Results:**

The pain detection threshold and pain tolerance of individuals with ASD were not impaired, indicating that there were no differences in the somatic perception of pain between groups. However, individuals with ASD were hyposensitive to subjective pain intensity compared to controls (VAS; electrical: *p* = 0.044, cold: *p* = 0.011, heat: *p* = 0.042) and hyposensitive to affective aspects of pain sensitivity (SF-MPQ; electrical: *p* = 0.0071, cold: *p* = 0.042).

**Conclusions:**

Our results suggest that the cognitive pathways for pain processing are impaired in ASD and, furthermore, that our methodology can be used to assess pain sensitivity in individuals with ASD. Further investigations into sensory abnormalities in individuals with ASD are needed to clarify the pathophysiologic processes that may alter sensory processing in this disorder.

## Background

Autism spectrum disorder (ASD) is a neuro developmental condition characterized by compromised social interactions, reduced verbal communication, stereotyped repetitive behaviors, and restricted interests [1]. The prevalence of ASD has increased to 1.0 % worldwide [2, 3]. However, the cognitive basis for ASD remains poorly understood and, as with other psychiatric disorders, the defining criteria are difficult to establish and measure objectively. The latest diagnostic criteria for ASD in the Diagnostic and Statistical Manual of Mental Disorders, 5th Edition (DSM-5) include the category of sensory abnormalities. However, no standard method exists to assess these symptoms in ASD. The ability to directly measure the degree to which sensory abnormalities exist in individuals with ASD would help to better define the severity of the disorder as well as the effectiveness of treatment options.

Sensory abnormalities have been described in individuals with ASD since the publication of the first clinical report [4]. The prevalence of abnormalities in sensory perception and cognitive processes has increased to 69–95 % among individuals with ASD [5–7]. These symptoms lead to problematic behaviors and maladaptation in the social and daily lives of these individuals [8]. Thus, abnormalities in sensory perception and cognitive processes are among the most important indications of ASD. The literature is inconsistent in determining whether ASD is characterized by hyposensitivity, hypersensitivity, or both [5]. No standard method exists for assessing sensitivity among individuals with ASD, and the neurological mechanisms underlying the observed abnormal sensory processing remain unknown [8, 9]. Therefore, this study was designed to establish useful methods for assessing sensory abnormalities and elucidating the characteristics of pain sensitivity associated with ASD.

Pain is defined as an unpleasant sensory and emotional experience associated with actual or potential tissue damage or described in terms of such damage [10]. Pain sensitivity is composed of somatic sensory perception and a subjective emotional reaction, and it plays a key role in warning people to avoid dangerous stimulation. However, relatively few studies pain processing have been conducted in individuals with ASD compared with the processing of other senses (e.g., auditory processing) [11]. C. S. Alley reviewed past studies that investigated pain among individuals with ASD and identified 15 studies among them [12]. Of those, five were case studies [13–17] and 10 were experimental [3, 4, 6, 18–24]. The five case studies explored pain perception, expression, or observer perception of pain in individuals with ASD [13–17]. One of these studies reviewed autobiographical accounts of pain sensing [14]. All of the case studies reported hyposensitivity among individuals with ASD [13–17]. The experimental studies, however, reported inconsistent results. One experimental study found no significant difference in pain intensity ratings between individuals with ASD and controls [18], two studies reported a high prevalence of pain hyposensitivity among individuals with ASD [3, 20], and three studies found that individuals with ASD experienced hypersensitivity to pain [4, 6, 19, 23]. Among these latter studies, one found that hypersensitivity to pain was associated with delayed diagnosis [21]. Another study reported that facial expression had a significant impact on the observers’ estimate of pain intensity, while the information on pain intensity did not [22]. The remaining study showed no significant correlation between CSF levels of beta-endorphins and clinical symptoms, including pain insensitivity [24].

These previous studies employed different types of stimulation and different assessment tools. Therefore, the inconsistent results may have been due to different methodologies. Moreover, these studies did not distinguish subjective pain sensitivity from sensory perceptions [12]. Previously, investigators relied on direct or indirect stimulation. Experimental stimulation with instruments detected sensory thresholds but did not detect subjective pain sensitivity [19]. Other examples of direct stimulation included venipuncture [4] and dental care [6]. The levels of administered stimulation also differed across participants. The indirect forms of stimulation applied in the previous reports depended on imagining painful situations [12]. Moreover, although pain is a subjective experience, most previous reports assessed pain sensitivity among individuals with ASD using observer reports [12].

To examine whether individuals with ASD have impaired sensory perception, subjective pain sensitivity or both, we first compared the pain detection thresholds and pain tolerance in these individuals compared with those of controls. Then, we defined the intensity at which each stimulation led to pain. Next, we compared the quantity and quality of subjective pain sensitivities to maximum stimulation levels between groups. Subjective pain sensitivities were assessed to avoid observer bias. We used two of the most popular assessment tools to evaluate subjective pain sensitivity: the visual analog scale (VAS) [25] and the short form of the McGill Pain Questionnaire (SF-MPQ) [26], both of which are brief. The VAS assesses subjective pain intensity, and the SF-MPQ provides data regarding pain intensity and pain type [26]. Thus, we assessed the characteristics of pain sensitivity among individuals with ASD.

## Methods

### Participants

The sample consisted of 15 patients with ASD and 15 healthy controls matched for age and sex. All participants provided written informed consent. The ethics committee at Osaka University approved this study, which was conducted in accordance with the World Medical Association’s Declaration of Helsinki.

### ASD assessment

We obtained data from patients with ASD from the research bio-resource of the Human Brain Phenotype Consortium in Japan (http://www.sp-web.sakura.ne.jp/consortium.html). Each patient with ASD was diagnosed by at least two trained child psychiatrists using the DSM-5 criteria. The participants were screened for comorbid psychiatric diagnoses and neurological disorders that might affect somatosensation. The diagnoses were based on unstructured or semi-structured behavioral observations of the patients as well as interviews with the patients and their parents or caregivers, as previously described [27]. In addition, the Autism Diagnostic Interview-Revised (ADI-R) [28], the Pervasive Developmental Disorders Autism Society Japan Rating Scale (PARS) [29], and the AQ-J [30] were used to evaluate ASD-specific behaviors and symptoms, as previously described [27]. Patients were recruited at Osaka University Hospital.

### Healthy controls

A previous report provided a detailed description of the healthy controls [31]. Controls were recruited through advertisements or silver centers. These participants were excluded if they had neurological, psychiatric, or chronic pain disorders. Individuals taking psychotropic or analgesic drugs during the course of the study were also excluded.

### Cognitive tests

Intelligence quotient (IQ) data of the individuals with ASD were collected using the full-scale Wechsler Adult Intelligence Scale-III [32] (*n* = 13), the full-scale Wechsler Intelligence Scale for Children-Third Edition [33] (*n* = 1), or the Japanese version of the NART 50, which can measure estimated IQ scores [34] (*n* = 1).

### Physiological stimulation

When participants could no longer endure the stimulus, stimulation was immediately stopped. Therefore, the methodology for pain tolerance was non-invasive and left no after-effects on the participants. The study was approved by the ethics committee at Osaka University. We obtained informed consent from all individuals regarding the methodology.

### Electrical stimulation

**Fig. 1:**
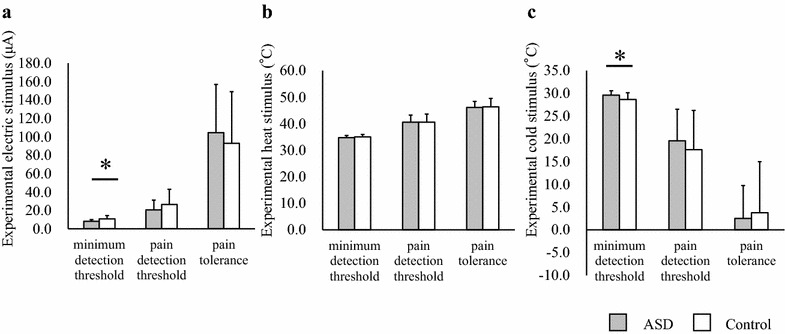
Comparison of somatic sensory thresholds between participants with ASD and controls. Differences in mean stimulation levels at the minimum detection thresholds, pain detection thresholds, and pain tolerance for electrical (**a**), heat (**b**), and cold (**c**) stimulations. *Error bars* represent SEs. *Symbols* represent the significance of *p* values. *p < 0.05. *ASD* autism spectrum disorder

The stimulation was performed with participants in a seated position in a quiet room at 16–22 °C. Each individual participated in the evaluation of pain thresholds using electrical stimulation. We used the Pain Vision System (PS-2100; Nipro Corporation, Osaka, Japan) [35], which was originally developed to estimate the pain intensity experienced by participants during gradual Aβ fiber stimulation. Approximately 5 min after placing the sensors and explaining the electric stimulation procedure to the participants, a gradually increasing amount of electrical stimulation was applied. The electrical stimulation was increased from 0 to 256 μA over a period of 1 min. Participants were instructed to push a button when they perceived pain, at which point, the stimulation was stopped. First, the minimum detection threshold (the point at which the participant first detected some sensation) was measured for each participant (Fig. 1a). Second, the pain detection threshold (the point at which the participants detected the electrical stimulation as pain) was measured (Fig. 1a). Finally, pain tolerance (the point at which the participants could no longer endure the pain) was measured (Fig. 1a).

### Thermal stimulation

The stimulation was performed with participants in a seated position in a quiet room at 16–22 °C. The thresholds for thermal (i.e., heat/cold) stimulation were evaluated for each individual. We applied graded heat/cold as a noxious stimulus and measured the maximum/minimum temperature that each participant was able to endure, as previously described [31]. Thermal stimulation was delivered accurately to the ventral surface of the non-dominant forearm via a 30 × 30 mm^2^ Peltier device (Pathway; Medoc Ltd, Ramat Yishai, Israel). This device was attached to the forearm with a strap and moved to an adjacent area after the presentation of every third stimulus to avoid habituation or sensitization to repeated stimulation. A baseline temperature of 32 °C was maintained. Stimulus temperatures were delivered at a rate of 1 °C/second and were feedback controlled. For safety purposes, this study limited the maximum/minimum stimulus temperature to 52/–10 °C. First, the warm/cold detection threshold (the point at which the participant perceived that the temperature was warm/cold) was measured for each participant (Fig. 1b, c). Second, the pain threshold (the point at which the participant experienced the temperature as pain) was measured (Fig. 1b, c). Finally, the heat/cold tolerance (the points at which the participant experienced the temperature as intolerable pain) was measured (Fig. 1b, c).

### Measurement of subjective pain sensitivity

**Fig. 2:**
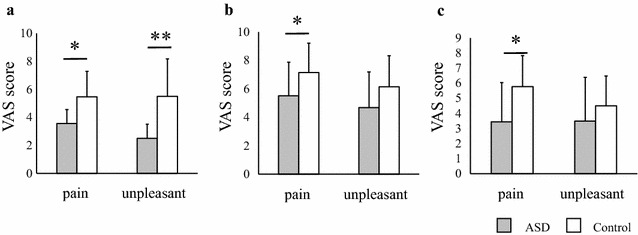
Comparison of subjective pain sensitivities assessed using the VAS between participants with ASD and controls. Differences in mean VAS scores with painful stimulations for two dimensions: pain and discomfort (**a** electrical, **b** heat, **c** cold). *Error bars* represent SEs. *Symbols* represent the significance of *p* values. *p < 0.05, **p < 0.01. *ASD* autism spectrum disorder

**Fig. 3:**
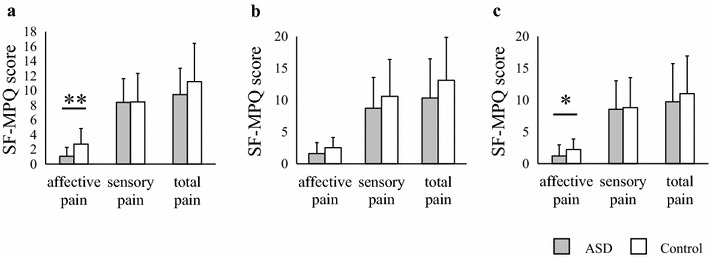
Comparison of subjective quality of pain sensitivity assessed by the SF-MPQ between participants with ASD and controls. Differences in mean SF-MPQ scores with painful stimulations for three dimensions: affective pain, sensory pain, and total pain (**a** electrical, **b** heat, **c** cold). *Error bars* represent SEs. *Symbols* represent the significance of *p* values. *p < 0.05, **p < 0.01. *ASD* autism spectrum disorders

To evaluate pain intensity, all of the participants were interviewed using the VAS (Fig. 2) and the SF-MPQ [26] (Fig. 3) to determine their pain tolerance (i.e., their intolerable pain stimulation level). As previously described, pain is defined as an unpleasant sensory and emotional experience [10]. Two different VASs were employed, one for pain intensity and one for discomfort. The VAS consists of a 100-mm linear intensity scale, where 0 = no pain and 10 = maximum pain imaginable. The SF-MPQ is composed of 15 items: items 1–11 represent sensory dimensions of pain experience, and items 12–15 represent the affective dimension of pain [26]. Each item is rated using an intensity scale, where 0 = none, 1 = mild, 2 = moderate, and 3 = severe [26]. The SF-MPQ provides three pain scores: total, sensory, and affective pain intensity. Participants were asked to choose the specific words (and their associated intensity scales) that best described their pain during their tolerable stimulus level. The VAS and SF-MPQ were administered to the participants immediately after stimulation.

### Statistical analysis

Statistical analyses were performed using SPSS (Statistical Package for the Social Sciences) for Windows version 16.0 software (SPSS Japan Inc., Tokyo, Japan). Group comparisons with regard to sex were performed using the *Χ*^*2*^ test. The Mann–Whitney *U* test was employed for comparisons of continuous variables, as appropriate. Between-group differences with regard to temperature (i.e., heat/cold stimulation), electrical stimulation, and VAS and SF-MPQ scores were analyzed using Mann–Whitney *U* tests. All *p* values reported are based on two-tailed tests. Statistical significance was defined as *p* < 0.05.

## Results

### Participant characteristics

The patients with ASD and healthy controls were matched for age (ASD 25.8 ± 9.2-year old, control 26.3 ± 7.5-year old, *U* = 103.0, *z* = −0.395, *p* = 0.7) and sex (male/female = 2/3). Additional characteristics of the individuals with ASD were as follows: education: 13.6 ± 2.6 years, full-scale IQ 101.8 ± 14.3, and total scores on the Japanese version of the Asperger’s Questionnaire (AQ-J; *n* = 11): 31.7 ± 7.4. These data were not available for the controls.

### Sensory thresholds

We measured the minimum detection threshold, pain detection threshold, and pain tolerance by having each participant push a button at each sensory point (Fig. 1). The minimum detection thresholds for electrical and cold stimulation occurred significantly earlier in the ASD group than in the control group (electrical: ASD mean ± standard error (SE) = 8.30 ± 1.71 μA, control = 10.87 ± 3.50 μA, *U* = 57.50, *z* = −2.28, *p* = 0.023; cold: ASD = 29.64 ± 0.93 °C, control = 28.66 ± 1.48 °C, *U* = 54.00, *z* = −2.43, *p* = 0.015; Fig. 1a, c). These results suggest that the ASD group was hypersensitive to electrical and cold stimulation. Alternatively, they may have responded to these stimulations by pushing the button earlier than the controls. The minimum detection thresholds for heat stimulation did not differ between the groups (ASD mean ± SE = 34.71 ± 0.82 °C, control = 34.99 ± 0.96 °C, *U* = 93.00, *z* = −0.81, *p* = 0.42; Fig. 1b). Therefore, the ASD group most likely pushed the button appropriately during the experiment. No significant differences were detected between groups with regard to the pain detection thresholds for each stimulation (electrical: ASD mean ± SE = 20.58 ± 10.72 μA, control = 26.44 ± 16.48 μA, *U* = 94.00, *z* = −0.77, *p* = 0.44; heat: ASD = 40.54 ± 2.68 °C, control = 40.56 ± 3.07 °C, *U* = 109.00, *z* = −0.15, p = 0.89; cold: ASD = 19.59 ± 6.92 °C, control = 17.60 ± 8.64 °C, *U* = 98.00, *z* = −0.60, *p* = 0.55; Fig. 1). In addition, no significant between-group differences were observed with regard to pain tolerance for each stimulation (electrical: ASD mean ± SE = 104.48 ± 52.23 μA, control = 92.86 ± 55.99 μA, *U* = 95.00, *z* = −0.73, *p* = 0.47; heat: ASD = 46.12 ± 2.23 °C, control = 46.35 ± 3.16 °C, *U* = 102.00, *z* = −0.44, *p* = 0.66; cold: ASD = 2.50 ± 7.22 °C, control = 3.74 ± 11.24 °C, *U* = 102.00, *z* = −0.44, *p* = 0.66; Fig. 1). These results suggest that the sensory thresholds for pain in the ASD group were not impaired.

### Subjective pain sensitivity measured using the VAS

The VAS pain scores for the ASD group were significantly lower than those for the control group for every stimulation (electrical: ASD mean ± SE = 3.55 ± 2.43, control = 5.47 ± 2.44, *U* = 64.00, *z* = −2.01, *p* = 0.044, Fig. 1a; heat: ASD = 5.51 ± 2.37, control = 7.15 ± 2.06, *U* = 63.50, *z* = −2.03, *p* = 0.042, Fig. 1b; cold: ASD = 3.44 ± 2.59, control = 5.77 ± 2.06, *U* = 51.00, *z* = −2.55, *p* = 0.011, Fig. 2c). For the electrical stimulation, the VAS discomfort score of the ASD group was lower than that of the control group (ASD mean ± SE = 2.50 ± 1.82, control = 5.50 ± 2.68, *U* = 44.50, *z* = −2.82, *p* = 0.0048; Fig. 2a). Although the mean discomfort scores for heat and cold stimulation within the ASD group were lower than those within the control group, significant differences were not found between the groups (heat: ASD mean ± SE = 4.69 ± 2.51, control = 6.15 ± 2.18, *U* = 75.50, *z* = −1.54, *p* = 0.13, Fig. 2b; cold: ASD mean ± SE = 3.49 ± 2.90, control = 4.50 ± 1.97, *U* = 80.50, *z* = −1.33, *p* = 0.18, Fig. 2c). These results suggest that the stimulations were less painful for the ASD group than for the controls (Fig. 2a–c). They also felt greater discomfort than the controls with regard to painful electrical stimulations. However, they felt the same amount of discomfort as the controls with regard to painful cold and heat stimulation. Therefore, subjective pain processing pathways in individuals with ASD may differ from those in controls.

### Subjective pain sensitivity measured using the SF-MPQ

The affective pain sensitivity scores associated with electrical and cold stimulation in the ASD group were lower than those in the control group (electrical: ASD mean ± SE = 1.07 ± 1.22, control = 2.73 ± 2.12, *U* = 49.50, *z* = −2.69, *p* = 0.0071, Fig. 3a; cold: ASD = 1.20 ± 1.74, control = 2.20 ± 1.66, *U* = 65.00, *z* = −2.03, *p* = 0.042, Fig. 3c). The mean score for affective pain sensitivity to heat stimulation was lower in the ASD group than in the control group; however, no significant differences were observed between the groups (ASD mean ± SE = 1.60 ± 1.72, control = 2.53 ± 1.60, *U* = 71.00, *z* = −1.76, *p* = 0.079; Fig. 3b). In contrast, the subjective pain sensitivity scores for the ASD group were not significantly different from those in the control group for any stimulation (electrical: ASD mean ± SE = 8.40 ± 3.22, control = 8.47 ± 3.87, *U* = 107.00, *z* = −0.23, *p* = 0.82, Fig. 3a; heat: ASD = 8.73 ± 4.83, control = 10.60 ± 5.79, *U* = 93.50, *z* = −0.79, *p* = 0.43, Fig. 3b; cold: ASD = 8.53 ± 4.49, control = 8.80 ± 4.72, *U* = 112.00, *z* = −0.021, *p* = 0.98, Fig. 3c). The mean total pain scores for the ASD group were lower than those for the control group (Fig. 3a–b). However, no significant difference in total pain was observed between the groups for any type of stimulation (electrical: ASD mean ± SE = 9.47 ± 3.56, controls = 11.20 ± 5.21, *U* = 86.50, *z* = −1.09, *p* = 0.28, Fig. 3a; heat: ASD = 10.33 ± 6.16, control = 3.13 ± 6.73, *U* = 84.50, *z* = −1.17, *p* = 0.24, Fig. 3b; cold: ASD = 9.73 ± 5.98, control = 11.00 ± 5.94, *U* = 95.00, *z* = −0.73, *p* = 0.47, Fig. 3c). These results suggest that the participants with ASD were impaired in their emotional evaluation of painful electrical and cold stimulations.

### Correlation between AQ scores and pain sensitivities

No significant differences were observed between the total AQ scores and any of the variables among individuals with ASD (data not shown).

## Discussion

To our knowledge, this is the first report to investigate sensory abnormalities related to pain in individuals with ASD using well-controlled stimulations. We distinguished physical sensory thresholds from subjective sensitivities to determine whether sensory abnormalities were based on the perception or recognition of certain stimulations. Then, we compared the subjective pain sensitivities between groups with regard to painful stimulations. We found that the sensory perception of pain was not impaired in individuals with ASD. However, individuals with ASD had hyposensitive subjective pain sensitivities compared to controls. These results suggest that individuals with ASD have impaired cognitive processing with regard to pain. This finding was most clearly observed with regard to the electrical stimulations. Using this method, we identified characteristics of pain sensitivity among individuals with ASD.

Significant between-group differences were observed with regard to the minimum detection thresholds for electrical and cold stimulations (Fig. 1a, c).

There are two possible causes for this latter observation. One possibility is that the differences were due to earlier detection in individuals with ASD. Another possibility is that individuals with ASD might push the button earlier than the controls, regardless of their detection threshold. This latter hypothesis is inconsistent with many reports that have suggested the presence of motor dyspraxia or clumsiness in individuals with ASD [36]. Furthermore, between-group differences did not exist in the minimum detection thresholds with regard to heat stimulation (Fig. 1b), suggesting that participants with ASD responded appropriately. These results suggest that individuals with ASD are hypersensitive to weak electrical and cold stimulations. Because our sample size was small, additional investigations with a larger sample size are warranted. The VAS scores for discomfort differed across stimulation types. Thus, the unpleasantness associated with strong stimulations among individuals with ASD differs across stimulation types.

Several tools are available to assess pain sensitivity in individuals with ASD. Sensory symptoms have often been evaluated in children with ASD using caregiver reports [8, 9]. One of the most common sensory measurements for individuals with ASD is the sensory profile (SP) [37]. Individuals with ASD might not express their pain sensitivity in the same way as typically developing individuals [12]. Individuals with ASD have less intense reactions than controls [3], which have been interpreted as indicating a lack of sensitivity [3, 22]. Thus, caregiver reports might be inaccurate representations of pain sensitivity in individuals with ASD. In addition, several self-reports have been created. The adolescent/adult SP (AASP), which was developed from the original SP [38], contains questions regarding other factors that might influence sensory experiences and affective reactions [39]. Both the AASP and the Sensory Over-Responsivity Scale have a similar problem [39], which is their dependence on the recall of sensitive situations. Therefore, these assessments can result in inaccurate answers because of ambiguous memories. Moreover, stimulation intensities are not always strong enough to cause pain, and they can differ across participants. To investigate pain in individuals with ASD, then, it is essential to evaluate both physical pain thresholds and subjective pain sensitivities using a sufficient number of stimulation types to ensure that pain has been evoked. Moreover, the assessment of pain itself is important. Our current methodology was able to resolve all of these problems.

Inconsistencies exist between this study and previous reports. One study reported that pain detection sensitivities for cold and heat stimulation were more sensitive among individuals with ASD than controls [19]. Because our groups included approximately twice as many participants as this study [19], these inconsistencies may have been due to difference in sample size.

We also measured the quality of pain sensitivity in this study. The affective aspects of subjective pain sensitivities of individuals with ASD were less intense than those of controls. Previously, the distress caused by sensory processing dysfunctions among individuals with ASD has led to self-injurious and aggressive behaviors [40]. Hypersensitivity is often correlated with increased anxiety [8]. Therefore, we hypothesized that the affective aspects of pain sensitivity would be high among individuals with ASD. On the contrary, these individuals reported lower levels of the affective aspects of pain than controls. However, individuals with ASD might have hypersensitive minimum detection thresholds. Our results suggest that the hypersensitivity to weak stimulations among individuals with ASD provokes strong reactions. Previous reports have shown that individuals with ASD have poor emotional control [41], which supports our findings.

Our study had several limitations. Our groups had a wide range of sensory evaluation results, which were likely due to the heterogeneity of the participants with ASD. Therefore, our results cannot fully explain the sensory processing of pain among these individuals. Because our sample size was small, additional studies are warranted using larger samples and various types of stimulation.

## Conclusions

Our results suggest that participants with ASD are hyposensitive to pain, and they recognize pain in a different way than control participants. The characteristics related to pain sensitivity might not be observed in all individuals with ASD. However, the current method is likely useful. Categorizing individuals with ASD by pain sensitivity levels might provide useful guidance for future investigations into the pathologic mechanisms contributing to this disorder. Because impaired sensory processing is not specific to individuals with ASD [42], comparisons of pain sensitivity with other neuropsychological diseases are warranted. The current findings may contribute to future investigations of the sensory features of ASD and clarify its pathophysiology.

## Abbreviations

ASD: Autism spectrum disorder
VAS: Visual Analog Scale
SF-MPQ: quality of pain assessed by the short-form McGill Pain Questionnaire
DSM-5: diagnostic and Statistical Manual of Mental Disorders, 5th Edition
SD: standard deviation
FIQ: full-scale intelligence quotient
ADI-R: Autism diagnostic interview-revised
PARS: pervasive Developmental Disorders Autism Society Japan Rating Scale
AQ-J: Japanese version of Asperger’s questionnaire
SE: standard error
